# Thermoresponsive Complex Coacervates as Advanced Carriers for Cell‐Laden Liquid‐Core Capsules for Biomedical Applications

**DOI:** 10.1002/smll.202513642

**Published:** 2026-04-16

**Authors:** Luís P. G. Monteiro, Mariana Carreira, Julien Es Sayed, Marleen Kamperman, João M. M. Rodrigues, João Mano

**Affiliations:** ^1^ CICECO – Aveiro Institute of Materials Department of Chemistry University of Aveiro Aveiro Portugal; ^2^ Zernike Institute for Advanced Materials University of Groningen Groningen The Netherlands

**Keywords:** bioinspired materials, complex coacervation, liquid capsules, polysaccharides, thermoresponsive

## Abstract

Injectable “smart” materials are emerging as promising platforms for minimally invasive cell delivery and tissue regeneration. A novel thermoresponsive complex coacervate was engineered through electrostatic interactions between natural polysaccharides grafted with poly(*N*‐isopropylacrylamide) (PNIPAAm). The resulting biopolymeric‐derived coacervate exhibits pronounced shear‐thinning behavior and undergoes a rapid sol–gel transition at physiological temperature. Rheological analysis revealed that the thermoresponsive PNIPAAm chains regulate network dynamics, with faster relaxation at 25°C and enhanced structuring at 37°C due to increased hydrophobic interactions. The designed complex coacervate provides an efficient transport vehicle for the in situ retention of liquid‐core capsules (LC) loaded with human adipose stem cells, promoting autonomous and hierarchical tissue organization. This system retained its shear‐thinning properties even at high LC volumetric ratios (up to 54%) and supported high cell viability at least for 7 days. This strategy enables cell encapsulation in a thermoresponsive injectable complex coacervate that can be loaded with virtually any, or even multiple, cell types, offering a highly modular and cytocompatible platform. Altogether, this work introduces a new paradigm for the design of bioinspired, thermoresponsive complex coacervates, offering hierarchical control over cellular organization enabling the decoupling of tissue‐forming units from the transport matrix.

## Graphical abstract

A thermoresponsive complex coacervate is engineered to transport liquid‐core capsules loaded with human adipose‐derived stem cells, forming modular, hierarchically organized tissue‐forming units. This injectable platform enables precise spatial control while maintaining high cell viability, offering a versatile and bioinspired strategy for minimally invasive tissue engineering and regenerative therapies.

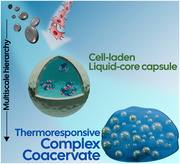

## 1 Introduction

Nature has provided us with a vast repertoire of molecular strategies that have inspired researchers to design advanced biomaterials [1, 2]. Among the vast group of bioinspired biomaterials reported in literature over the last decades, the paramount importance of complex coacervation has only recently begun to be unveiled [3, 4, 5]. Complex coacervation is a liquid‐liquid phase separation (LLPS) phenomenon driven by noncovalent interactions, but primarily through electrostatic interactions between two oppositely charged (macro)molecules [5, 6]. In nature, LLPS is ubiquitous [7, 8], from membraneless organelles such as nucleoli, Cajal bodies, and RNA granules [9], to the adhesive mechanisms of sandcastle worms and mussels [10, 11]. This highlights how a fine thermodynamic balance between coexisting liquid phases can be crucial for achieving specific biological functions. Complex coacervation results in the formation of a two‐phase system: a dense polymer‐rich and a polymer‐depleted diluted phase. The former describes a concentrated phase of associated oppositely charged polyelectrolytes (PE), known as the complex coacervate phase, whereas the latter contains only vestigial amounts of the PE [6, 12, 13]. Complex coacervation is a powerful mechanism that has the potential to enable the development of several types of biomaterials such as artificial cells [14, 15], medical adhesives [13, 16], cell‐loaded hydrogels [17], drug delivery systems [18, 19], micelles [20], biomaterial inks [3], among others [21]. Despite numerous reports on in situ crosslinked hydrogels [22], particularly those triggered via thermoresponsive gelation [23, 24, 25, 26, 27, 28], the development of complex coacervate‐based biomaterials, incorporating thermoresponsive domains that triggers a sol–gel transition, has significantly lagged behind. The combination of complex coacervation with thermoresponsive domains holds tremendous potential as an in situ forming material, an adhesive, a biomaterial for tissue regeneration, or a drug release platform, since it can be easily injected into the target site and effectively manipulated, due to its water‐immiscible properties [29]. Furthermore, no chemical reaction occurs during the sol–gel transition, ensuring that the elimination of the components by the bloodstream is not a concern. Importantly, this transition occurs within a short time and is triggered by the body's physiological temperature. These features enable the material to seamlessly adapt to geometrical irregularities of the target site, making it a versatile and efficient solution for biomedical applications [30]. Recently, the first thermoresponsive complex coacervate was reported based on synthetic PE grafted with Poly(N‐isopropylacrylamide) (PNIPAAm) chains, a well‐known thermoresponsive polymer, to serve as an underwater adhesive [13]. Despite the ease of chemical manipulation offered by synthetic polymers, natural polysaccharides offer a unique set of advantages, including their origin from renewable resources, high biodegradability, and low toxicity [31, 32]. Other strategies have explored PNIPAAm‐based hydrogels based on covalently bonded natural polysaccharides [33]. However, its low pre‐injection viscosity and cell anti‐migration properties may result in insufficient material retention within a defect, limiting cell incorporation and tissue regeneration potential.

Herein, we introduce a thermoresponsive complex coacervate system assembled through electrostatic interactions between chitosan (CHT)‐*g*‐PNIPAAm and hyaluronic acid (HA)‐*g*‐PNIPAAm. We hypothesize that these naturally derived coacervates offer a versatile and straightforward platform for the efficient transport of biological materials and hold great potential to be used in advanced biomaterials applications.

## 2 Results and Discussion

**FIGURE 1:**
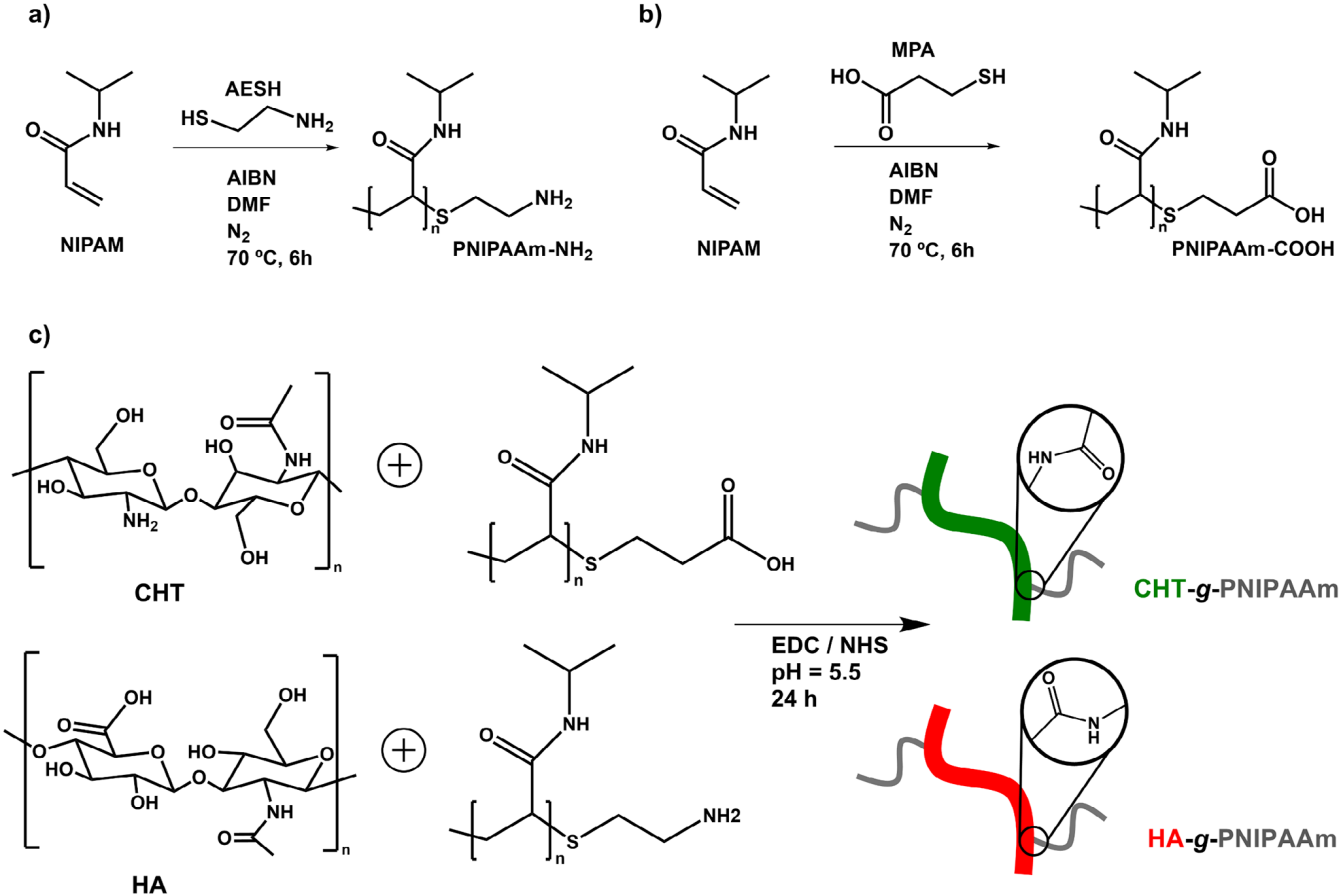
Chemical routes to obtain PNIPAAm‐NH_2_ and PNIPAAm‐COOH, as well as CHT‐*g*‐PNIPAAm and HA‐*g*‐PNIPAAm. Polymerization of NIPAAm bearing ─NH_2_ (a) and ─COOH (b) end groups. (c) Carbodiimide‐mediated grafting of PNIPAAm chains onto both CHT and HA backbones.

Thermoresponsive polymers have gained significant attention in recent years due to their ability to impart a “smart” trigger to the material [34]. Natural PE combined with thermoresponsive polymers offers the potential to design nature‐based, extracellular matrix–mimicking biomaterials. PNIPAAm is a synthetic, FDA‐approved [35], thermoresponsive polymer with a lower critical solution temperature (LCST) around 32°C [36, 37], which, during its polymerization, can yield chains bearing reactive end groups that enable subsequent coupling to both HA and CHT. The polymerization of both PNIPAAm‐NH_2_ and PNIPAAm‐COOH, as illustrated in Figure 1a,b, respectively, was confirmed by proton nuclear magnetic resonance (^1^H NMR) spectroscopy (see Figures S1a and S2a, respectively).

Analysis of the ^1^H NMR spectra of both modified compounds reveals a characteristic signal at 1.13 ppm, assigned to the six protons of the isopropyl group (‐(C**H_3_**)_2_), and a signal at 3.89 ppm corresponding to the methine group (─NH‐C**H)**. The introduction of the chain transfer agents, 2‐aminoethane‐1‐thiol (AESH) or 3‐mercaptopropionic acid (MPA), was confirmed by the signals observed between 2.60 and 3.00 ppm. The broad peaks at 2.00 and 1.57 ppm are attributed to the methylene (C**H_2_**) and methine (C**H**) protons of the chain backbone, with an integration ratio of 2:1, respectively. Additionally, DMF‐based size‐exclusion chromatography (DMF‐SEC) was employed to analyze the number‐average molecular weight (*Mn*), weight‐average molecular weight (*Mw*), and polydispersity index (PDI) of the polymers (see Figures S1b and S2b). PNIPAAm‐NH_2_ exhibited *Mn* and *Mw* values of 19,900 Da and 41 100 Da (PDI = 2.06), respectively, while PNIPAAm‐COOH showed *Mn* and *Mw* values of 24,800 Da and 47 000 Da (PDI = 1.89), consistent with broad but uniform chain populations. Upon the successful polymerization of the modified PNIPAAm chains, EDC/NHS coupling chemistry was employed to graft the synthetic polymers onto the CHT and HA backbones (Figure 1c). The ^1^H NMR spectra (see Figures S3 and S4) of both PE grafted with PNIPAAm showcase characteristic signals of the synthetic polymer, particularly at 1.13 ppm (C**H_3_** groups), confirming successful coupling. The molar ratios of PE repetitive units to NIPAAm were 60:40 for HA and 70:30 for CHT. The obtained grafting ratios are similar to those reported in previous studies [13] and were shown to yield a good balance between electrostatic interactions and thermoresponsive behavior.

### 2.1 Optimization of the Coacervate Assembly

**FIGURE 2:**
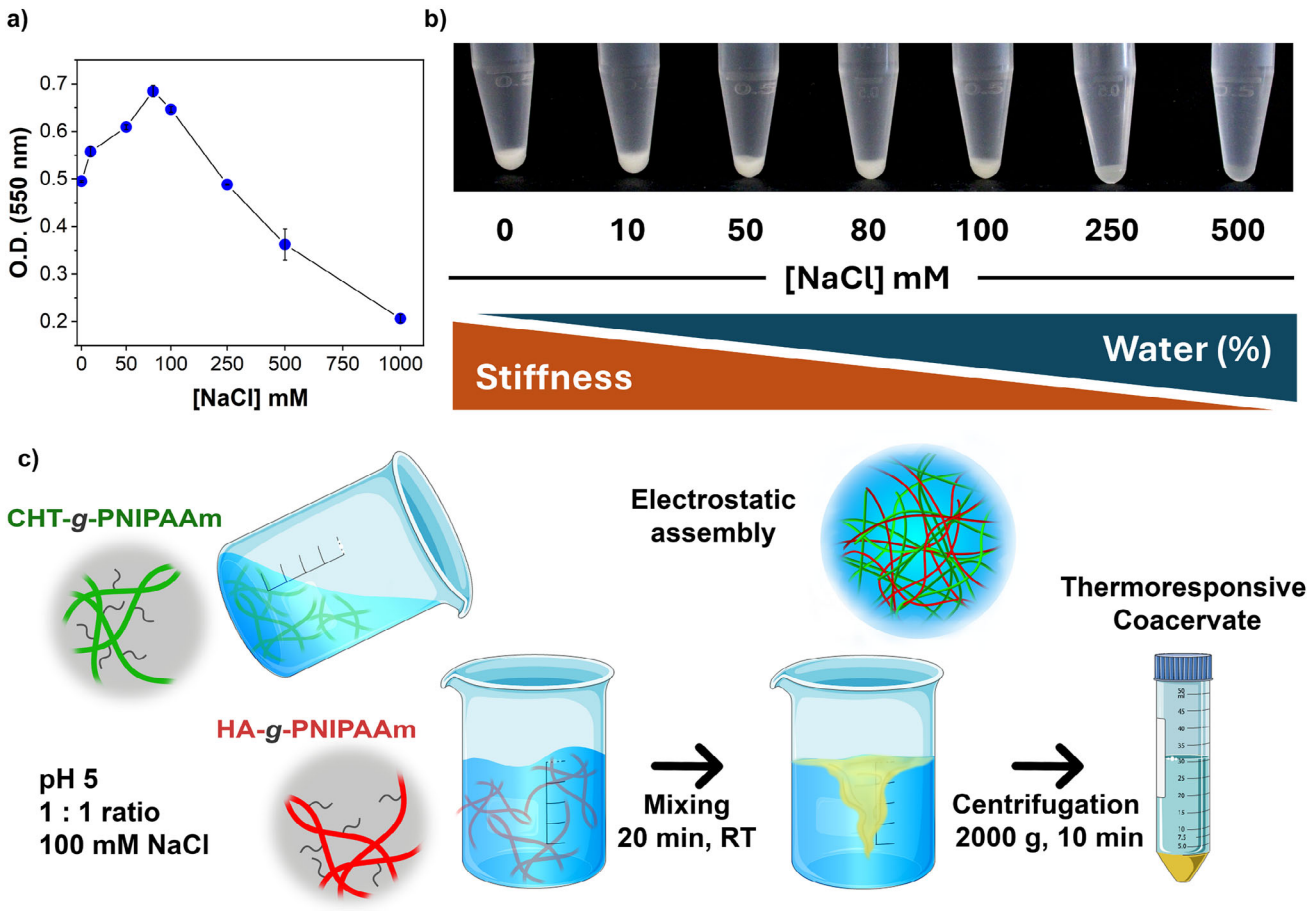
Optimization and assembly conditions of the CHT‐*g*‐PNIPAAm / HA‐*g*‐PNIPAAm thermoresponsive complex coacervates. (a) Optical density (OD) measurements of the coacervates at different NaCl concentrations; (b) corresponding digital images of the coacervates prepared with different NaCl concentration after centrifugation; and (c) schematic illustration of the procedure used to assemble the thermoresponsive coacervates. Icons from BioRender.

To develop an effective thermoresponsive coacervate, it is crucial to determine that the LCST of the PE‐*g*‐PNIPAAm conjugates remains below 37°C, consistent with the intrinsic PNIPAAm behavior [38]. The LCST was studied by measuring the transmittance of polymer solutions with increasing temperatures (Figure S5a). Both PNIPAAm‐NH_2_ and – COOH display an LCST around 34°C, which aligns with the typical LCST of PNIPAAm (32°C) [36, 38, 39]. Upon grafting PNIPAAm onto both HA and CHT, a small increase in the LCST to approximately 35≈36°C, respectively, was observed. This shift is attributed to the increased hydrophilicity granted by the PE backbone, which enhances polymer‐water interactions and delays the dehydration‐driven phase transition [40, 41]. The coacervate assembly is strongly influenced by several parameters such as pH, mixing ratio, and ionic strength, which are critical for understanding the precise design and specific application of coacervate‐based systems, playing crucial roles in modulating the coacervation process [42]. Determining the optimal mixing pH is fundamental to ensure that both PE possess the highest degree of ionization, thereby enhancing the electrostatic interactions and maximizing the coacervates yield. Zeta potential measurements were conducted at different pH values for both PE (Figure S5b,c), revealing that the strength of electrostatic interactions (SEI) was maximal around pH 5. Furthermore, the optimal mixing ratio, where no excess charge is detected in the diluted phase of the coacervate, was determined by measuring the zeta potential of the diluted phase in various PE mixtures at different mass ratios. A 1:1 mixing ratio was determined to be optimal, as negligible excess charge was detected in the diluted phase (Figure S5d), showcasing effective electrostatic charge balance within the coacervate phase. Lastly, salt concentration is another important parameter influencing the viscoelastic behavior and yield of the coacervate phase [43]. Optical density (OD), which can be directly correlated with turbidity, is commonly used to study the effect of salt on coacervate formation, as it reflects changes in phase separation [44, 45], with higher OD values indicating increased turbidity and, consequently, enhanced complexation. As shown in Figure 2a, low salt concentrations initially promoted coacervate formation, reflected by an increase in OD, the partial screening of electrostatic interactions facilitates chain mobility and rearrangement [46], ultimately favoring the formation of larger coacervate domains, a common behavior in complex coacervation [47, 48]. Maximum OD values were observed with 80 and 100 mm of NaCl, with no apparent differences in the amount of coacervate. At higher salt concentrations a pronounced decrease in OD was observed, ultimately leading to the suppression of coacervation (critical salt concentration), since most of the PE charges are screened by the salt ions. To better recreate the physiological conditions, and since 100 mm NaCl falls within the physiological range of salt concentrations in the human body [8, 49], this concentration was selected for the complex coacervate assembly.

These conclusions were also visually assessed, as described in Figure 2b, where it can be observed that at 500 mm of NaCl almost no coacervate was formed. Additionally, a closer observation revealed an increase in transparency of the coacervate phase as the NaCl concentration increases, which is linked to the higher water content caused by a reduced PE content in the coacervate phase [3, 50, 51]. Having identified the optimal conditions for electrostatic assembly, pH 5, a 1:1 mass ratio, and 100 mm of NaCl, the coacervates were assembled as depicted in Figure 2c.

### 2.2 Water Content Evaluation and Rheological Characterization

**FIGURE 3:**
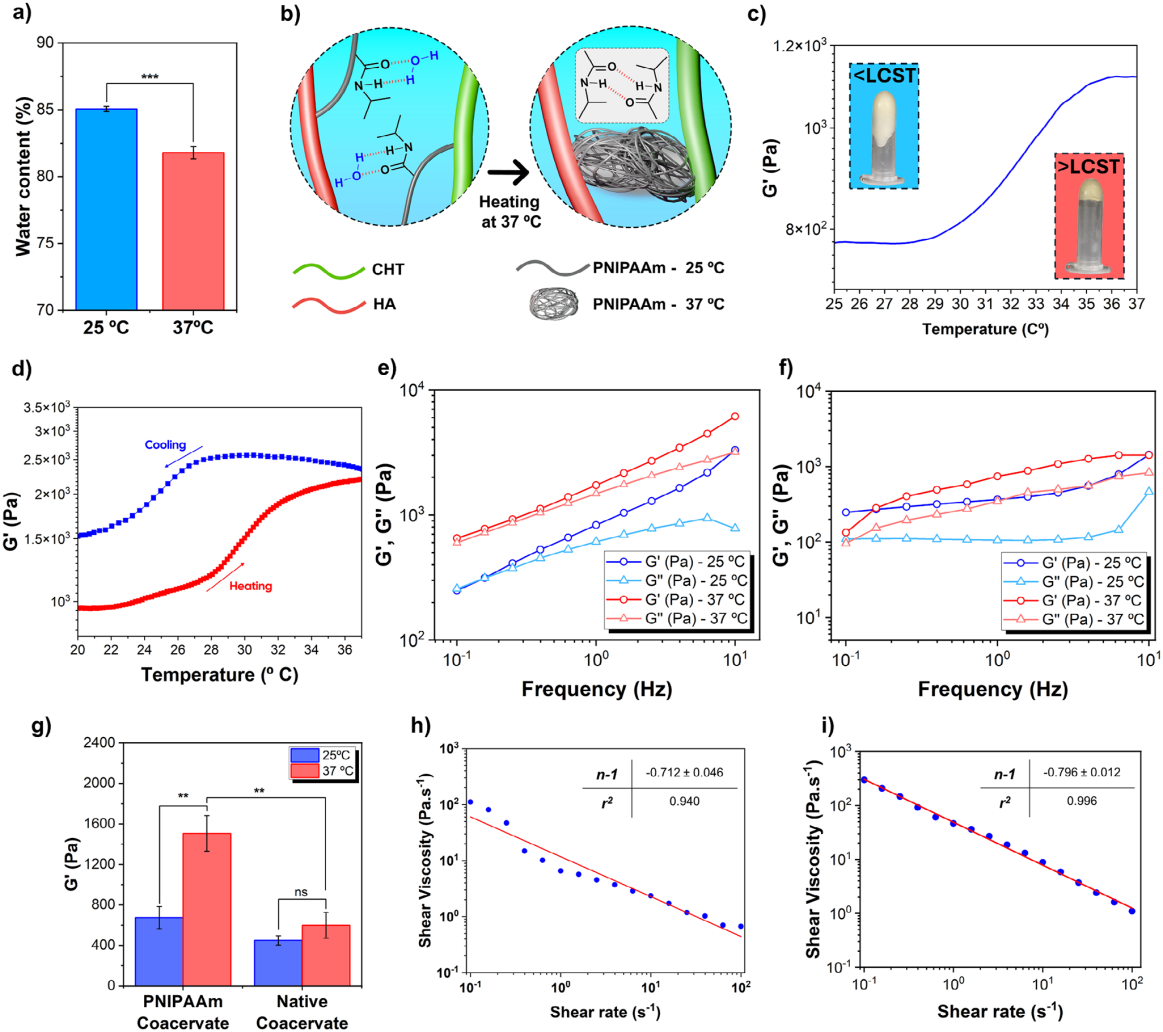
Impact of the thermal structuring of the thermoresponsive coacervate. (a) Water content of the thermoresponsive coacervates at 25°C and 37°C (*n* = 3); (b) Schematic illustration of the temperature‐dependent behavior of PNIPAAm chains below and above the LCST; (c) Storage modulus (G′) profile during a temperature sweep from 25°C to 37°C; (d) Heating/cooling cycle performed at 1 Hz, 0.1% strain, with a rate of 2.0°C min^−^^1^ for the thermoresponsive coacervates. Red squares correspond to the heating step, while blue squares represent the cooling step; Frequency sweep analysis of G′ and loss modulus (G″) at 25°C and 37°C for the (e) thermoresponsive and (f) native coacervates at 0.1% strain; (g) Comparative G′ values at 1 Hz for both coacervate systems (*n* = 3); Shear‐thinning behavior and corresponding power‐law fitting for the h) thermoresponsive and (i) native coacervates. *p* ≤ 0.05 (*), *p* ≤ 0.01 (**), *p* ≤ 0.001 (***), while ns denotes no statistically significant difference.

To further assess the thermoresponsive behavior of the coacervates, we studied their hydration state and rheological properties in response to an increase in temperature. To evaluate how temperature modulates the composition of the coacervates, the water content at both 25°C and 37°C was gravimetrically assessed (Figure 3a). The results demonstrate a significant decrease in the water content of the coacervate when heated at 37°C, a tendency frequently observed in PNIPAAm hydrogels [52, 53]. This behavior is a direct consequence of the volume phase transition of PNIPAAm chains above its LCST, as illustrated in Figure 3b. Below the LCST, the polymer chains are highly hydrated due to the presence of multiple hydrogen bonding sites (C═O and N─H), which enable water molecules to solvate the polymer in its random coil conformation [54, 55]. However, at higher temperatures, the PNIPAAm side chains interact with each other via protein‐like hydrogen bonds (C═O∙∙∙H─N) [56] and through hydrophobic interactions involving the isopropyl groups [57, 58], triggering a conformational transition from soluble coil structures to insoluble globules [59]. This process ultimately leads to a reduction in the number of bound water molecules [54, 59, 60]. To further assess whether this thermally induced process translates into macroscopic changes, the swelling behavior and macroscopic shrinkage of the thermoresponsive coacervates at 37°C were evaluated over time (Figure S6). As shown, the coacervates exhibited a gradual deswelling, reaching −23.2 ± 3.0% after 60 min of incubation. Interestingly, this reduction in swelling was not accompanied by a significant macroscopic shrinkage of the coacervate, as the area remained largely unchanged (∼98% of the initial area) after 60 min at 37°C. These results suggest that, although water is partially expelled from the system, the overall macroscopic dimensions of the coacervate are largely preserved. A similar behavior has been previously reported for PNIPAAm‐functionalized complex coacervates [61].

This behavior can also be evaluated through rheology to determine whether the thermally induced changes in the coacervates translate into mechanical responses [13]. To this end, a temperature sweep was performed to monitor the evolution of the storage modulus (G′) as a function of temperature – see Figure 3c. The G′ starts increasing around 28°C, indicating the onset of a thermally driven network reinforcement associated with the PNIPAAm phase transition, as described above. A plateau was reached just below physiological temperature, corresponding to an overall increase of approximately 350 Pa, suggesting that the body's natural temperature is sufficient to fully trigger the gelation process. For comparison, a time‐sweep test at 37°C was performed using both the native (defined as a coacervate assembled from the native polyelectrolytes, i.e., without grafted PNIPAAm) and the thermoresponsive coacervate (Figure S7a). As expected, the native coacervate showed no significant changes in G′ over time, confirming the absence of thermally induced structural organization. In contrast, the thermoresponsive coacervate exhibited an immediate increase in G′, followed by a gradual rise that slowed after *ca*. 8 min. This behavior highlights the fast gelation kinetics driven by PNIPAAm chains and suggests that structural reorganization at 37°C occurs on a relatively short timescale, which is characteristic of PNIPAAm‐based systems [62]. Furthermore, the thermoreversibility of the coacervate was assessed using heating–cooling cycles (see Figure 3d). As already demonstrated, G′ increases with temperature. During the cooling step, performed at the same rate (2°C/min), the G′ decreases, however, it does not overlap with the heating curve. The resulting hysteresis loop indicates that the rheological behavior during cooling is distinct from the one during heating [63, 64]. Such hysteresis is driven by the temperature‐dependent differences in the interchain associations (heating) and dissociation (cooling) between the hydrophobic N‐isopropyl groups of PNIPAAm, which display different association/dissociation kinetics [63, 65, 66]. The linear viscoelastic region (LVER) of the coacervates was initially established (Figure S7b), after which the linear viscoelasticity of the coacervates was characterized by analyzing the G′ and G″ over a defined frequency range (0.1–10 Hz), at both 25°C and 37°C. The thermoresponsive coacervate exhibited significantly different responses at the two tested temperatures (Figure 3e). At 25°C, G′ was higher than G″ across most of the frequency range, however, a crossover occurred at lower frequencies (0.16 Hz), where G″ becomes higher than G′, corresponding to a relaxation time [67, 68] (τ_R_) of 6.25 s. This behavior is characteristic of a viscoelastic liquid and has been observed in other complex coacervates [69]. Furthermore, in a stress‐relaxation test at constant strain (Figure S7c), the coacervate exhibited a half‐relaxation time of 2.54 ± 0.16 s, with more than 90% of the stress relaxed within the first 100 s, indicating rapid energy dissipation. These results indicate that the coacervate can easily flow under applied stress and rapidly adapt its shape after injection [70, 71]. At 37°C, the coacervate exhibits less frequency dependence, with G′ consistently higher than G″ across the entire frequency range, indicating elasticity‐dominated networks characteristic of a gel‐like behavior [72]. This behavior reflects the conformational transition of PNIPAAm chains across the LCST. At 25°C, the chains confer hydrophilicity to the system, enhancing the coacervate solvation by facilitating water‐polymer interactions and thus accelerating the relaxation dynamics. In contrast, at 37°C, the coil‐to‐globule transition increases hydrophobicity, leading to slower relaxation kinetics that fall outside the detectable frequency range [73, 74, 75]. In contrast, the coacervates lacking PNIPAAm exhibit gel‐like behavior at both at 25°C and 37°C, with G′ consistently higher than G″ and minimal frequency dependence (Figure 3f). This behavior is in good agreement with previously reported results on HA–CHT coacervates (pH 6, 100 mm NaCl) [69]. The differences between native and thermoresponsive coacervates at 25°C can be attributed to the lower availability of ionizable groups in the latter, as these are partially screened by the grafted PNIPAAm chains. Additionally, the presence of PNIPAAm chains renders a more liquid‐like behavior, as their hydration is enhanced in this regime. The influence of temperature on the mechanical strength of the coacervate was also evaluated by comparing the G′ values at 1 Hz for both native and thermoresponsive coacervate (Figure 3g). An increase in temperature from 25 to 37°C resulted in a significant increase in G′ for the thermoresponsive coacervate, whereas no statistically significant differences were observed for the native coacervate. These results further confirm that the mechanical reinforcement of the coacervate is triggered by the activation of the thermoresponsive domains. These results highlight the thermally induced structuring of the thermoresponsive coacervates, where the collapse of PNIPAAm chains at 37°C, by reducing the water content, promotes a denser packing of the PE chains, as illustrated in Figure S7d. However, as previously demonstrated, heating–cooling cycles reveal that during the cooling step the material does not fully recover its initial viscoelastic properties within the experimental timeframe. To further evaluate this recovery over extended periods, the coacervate was maintained at 37°C for 30 min and then allowed to equilibrate at 25°C for 1 h, after which its viscoelastic response was analyzed via a frequency sweep test (Figure S7e). The results show that the coacervate largely recovers its initial viscoelastic behavior, with G′ and G″ values comparable to those of the freshly prepared material, maintaining a viscoelastic liquid‐like behavior.

Furthermore, to investigate the flow behavior of the coacervates under applied shear force, the shear viscosity was measured across increasing shear rates. Both thermoresponsive (Figure 3h) and native (Figure 3i) coacervates exhibited non‐Newtonian shear‐thinning behavior, as the shear viscosity decreased with increasing shear rates. Additionally, a power‐law model was fitted to the experimental data to calculate the flow index (*n*). For both the thermoresponsive and native coacervates, the *n* values were 0.288 and 0.204, respectively, confirming their non‐Newtonian behavior, an essential feature for the development of injectable systems [76]. These values are in good agreement with previously reported flow indices for CHT‐HA coacervates [3].

### 2.3 In Vitro Biological Performance

**FIGURE 4:**
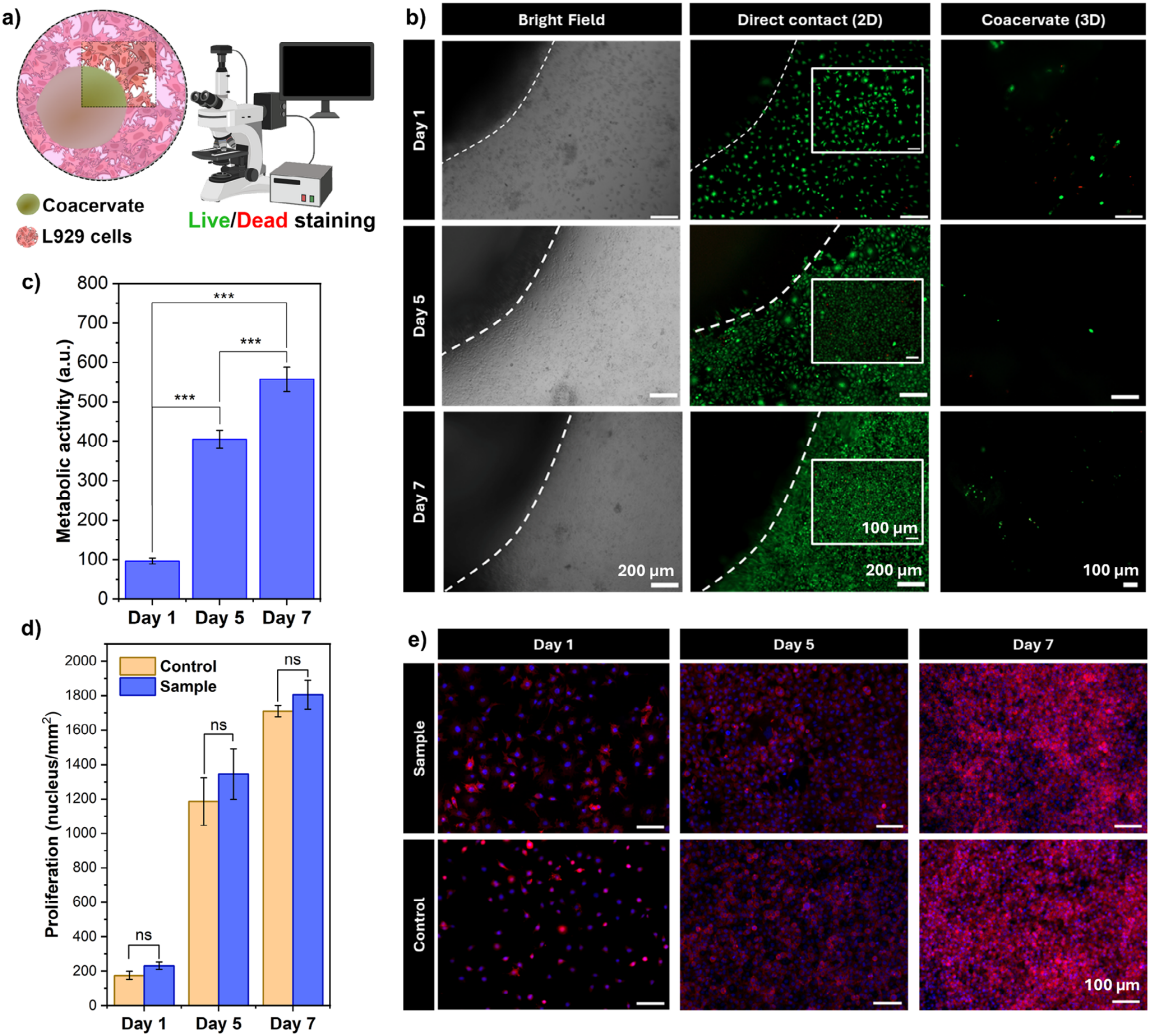
Cytocompatibility studies of the developed coacervates. (a) Schematic representation of the experimental setup used for direct contact assays between the coacervates and L929 cells. (b) Live/dead fluorescence microscopy images of L929 cells cultured in the presence of the coacervates. The panel includes bright‐field images, 2D direct contact conditions (cells adhered to the culture plate), and 3D coacervate contact conditions (cells in direct contact with the coacervate material). Staining includes Calcein AM (green, live cells), propidium iodide (PI, red, dead cells), and bright‐field analysis. (c) Quantitative analysis of cellular metabolic activity at the corresponding time points, (*n* = 3) *p*‐value ≤0.001 (***). (d) Corresponding cell proliferation analysis, expressed as cell density (cells/mm^2^), determined by image quantification using ImageJ. (e) Fluorescence microscopy images of cells stained with DAPI (blue, nuclei) and phalloidin (red, F‐actin cytoskeleton) at the same time points. Icons from BioRender.

To evaluate whether the coacervates exert any cytotoxic effects on neighboring cells, a cytotoxicity assay using L929 fibroblasts was performed. Cells were first cultured for 24 h in 24‐well plates, after which the coacervate was directly placed on top of the cell monolayer (Figure 4a). Cell viability and proliferation were then assessed at multiple time points. Live/dead staining revealed high cell viability with no signs of cytotoxicity over the 7‐day culture period (Figure 4b). In parallel, metabolic activity measured by Alamar Blue assay increased significantly from day 1 to day 7 (Figure 4c), suggesting enhanced cell proliferation. To further support these findings, cell proliferation was quantified using DAPI‐stained images, with cell density (cells/mm^2^) determined by nuclear counting through ImageJ analysis (Figure 4d). The results confirmed a consistent increase in cell proliferation over time, in line with the metabolic activity trend. This favorable cell response behavior, observed across live/dead staining, metabolic activity, and proliferation assays, was comparable to that of the control group cultured without coacervate exposure (Figure S8). Moreover, Phalloidin staining demonstrated that the coacervate had no harmful impact on cell morphology throughout the 7 days of culture (Figure 4e). Upon contact with culture medium, the coacervate equilibrates to physiological pH (∼7.4), which ensures that direct contact with the cell monolayer does not negatively impact cell survival, proliferation, or morphology, underscoring its biocompatibility and supporting its potential safe application in tissue defect environments without cytotoxic effects on surrounding cells. Considering that complex coacervation is a highly pH sensitive phenomenon, it is important to assess how the viscoelastic properties of the coacervate change after equilibration to physiological conditions.

**FIGURE 5:**
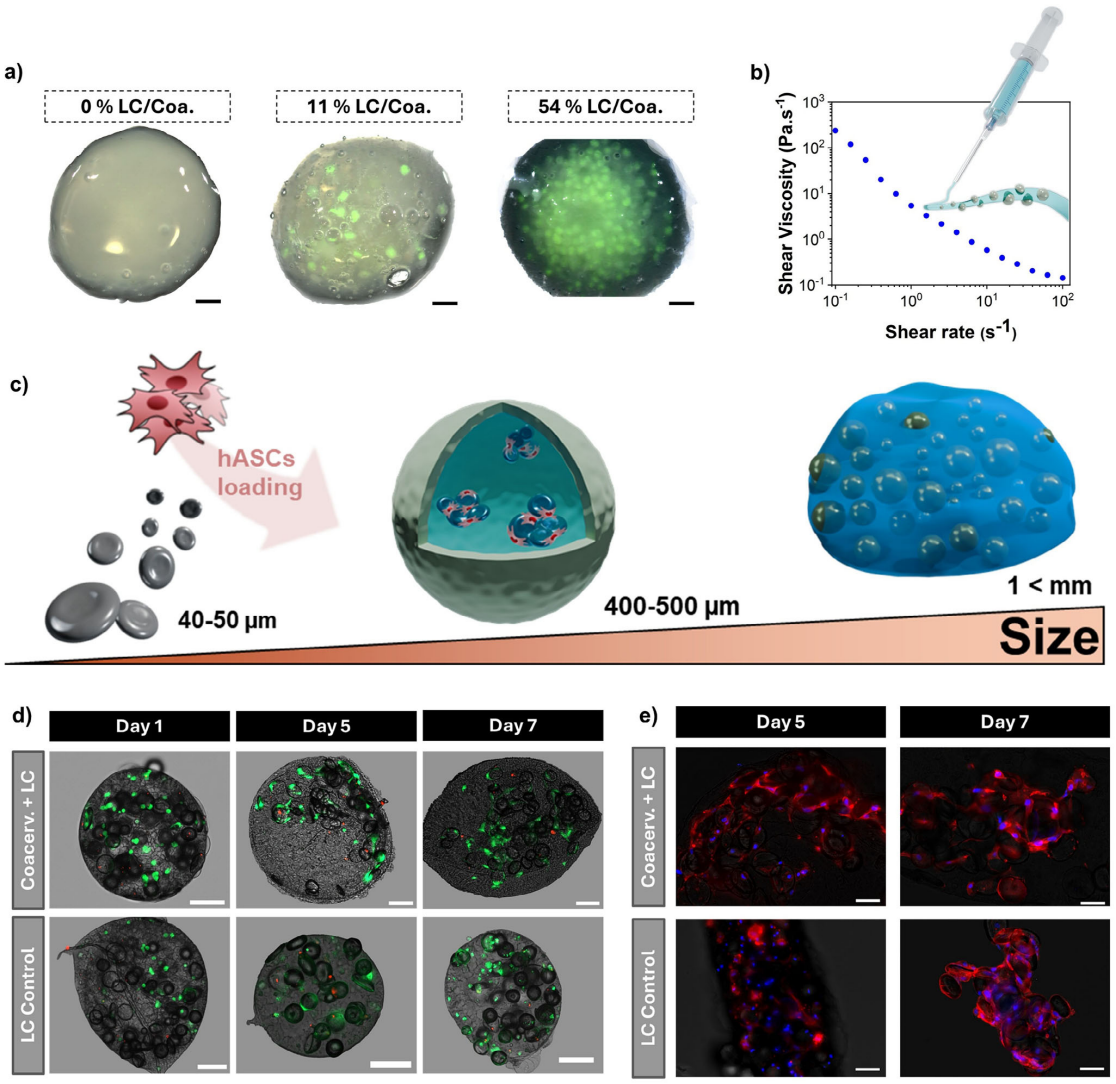
Structural, rheological, and biological assessment of LC‐loaded thermoresponsive coacervates. (a) Coacervates (80 µL) containing varying volumetric ratios of LC, scale bar = 1000 µm. For visualization purposes, the PCL microparticles were stained with coumarin 6; (b) Shear‐thinning profile of coacervates containing 54% LC; (c) Hierarchical size organization of the injectable coacervate system, where collagen‐coated PCL microparticles serve as adhesion substrates for hASCs within LC, which are later dispersed throughout the thermoresponsive complex coacervate matrix; (d) Live/Dead (scale bar = 100 µm) and (e) DAPI/phalloidin (scale bar = 50 µm) fluorescence staining of hASCs within the LC after being isolated from the coacervate at predetermined time points. Icons from BioRender.

To this end, the coacervate was incubated in PBS (pH 7.4), and its viscoelastic response was evaluated through a frequency sweep test (Figure S9). Despite the moduli values remaining comparable to those of freshly prepared coacervates, the coacervates incubated in PBS exhibit a gel‐like behavior (G′ > G″) across the entire frequency range, whereas freshly prepared coacervates exhibit a viscoelastic liquid‐like behavior. This is consistent with previous reports showing that HA–CHT coacervates formulated at pH ≥ 6 typically exhibit gel‐like behavior due to long‐lived cooperative hydrogen bonds within the coacervate network [69, 77]. In other words, at this pH, the dynamics of the coacervate are no longer fully governed by electrostatic interactions. When designing a system to be delivered in situ, and ultimately promoting tissue regeneration, the ability to incorporate stem cells is of paramount importance. To this end, human adipose tissue mesenchymal stem cells (hASCs) were directly encapsulated within the designed thermoresponsive coacervate, and cell viability was assessed. As expected, the live/dead assay revealed substantial cell death following encapsulation (Figure S10). The prolonged exposure of the encapsulated cells to the coacervate's slightly acidic pH, combined with the lack of adhesion cues for the anchorage‐dependent hASCs, compromises cell viability and proliferation during encapsulation. This observation is consistent with the well‐recognized limitation of CHT‐based materials regarding their capacity to accommodate encapsulated cells [78, 79, 80]. To overcome this drawback, we employed a cell encapsulation platform previously reported by our group (Figure S11a) [81], in which hASCs are encapsulated with polycaprolactone (PCL) microparticles within semipermeable liquid‐core capsules (LC). These LC, with an average diameter of 469 ± 46 µm (Figure S11b), are formed by interfacial complexation of oppositely charged polyelectrolytes in an all‐aqueous two‐phase system using electrohydrodynamic atomization equipment. We envision that the coacervates can act as a thermoresponsive vehicle for the transport and fixation of these biologically active hybrid LC into specific defected sites. This configuration enables precise delivery and mechanical integration of the LC at defect sites, while ensuring the perfect decoupling between the cellular microenvironment and the external matrix, a key concept in the engineering of hierarchical systems that mimic the modularity and compartmentalization of native tissues [82]. We first investigated the incorporation of LC into the coacervate matrix with increasing volumetric fractions. Coacervates were fabricated as described above, and predetermined volumetric fractions (%) of LC were then homogeneously mixed into the coacervate. As shown in Figure 5a, the coacervate retained its macroscopic integrity even when LC occupied up to 54% of its total volume, highlighting the robustness of the coacervate structure and its ability to withstand high LC loadings without disintegration or loss of cohesion. Furthermore, since the incorporation of LC could potentially affect the coacervate´s injectability, we evaluated its shear‐thinning behavior at the highest LC % loading (Figure 5b). As the shear rate increased, shear viscosity decreased, displaying a similar profile to that observed for coacervates without LC (Figure 3h). Having confirmed that LC incorporation does not compromise the injectability and cohesion of the coacervate matrix, we next investigated the capacity of the complex coacervate to function as a transport vehicle for the cell‐laden LC while preserving hASC viability. As illustrated in Figure 5c, collagen‐coated PCL microparticles act as adhesion substrates for hASCs within the LC, yielding modular micro‐scale tissue‐forming units. These units are subsequently embedded within the thermoresponsive coacervate, resulting in a multiscale, multimaterial hierarchy construct.

Initially, LC loaded with hASCs were cultured under agitation in a spinner flask for 24 h to promote cell adhesion to the PCL microparticles. Given the anchorage‐dependent nature of hASCs, the presence of PCL microparticles provide essential adhesion cues, thereby supporting effective cell attachment, proliferation, and functional maintenance within the liquid core of the capsules. This strategy enables cell encapsulation within the coacervates in an already adherent fashion (see Figure S12). The live/dead staining (see Figure S13) of the LC loaded into the coacervate revealed that the hASCs remained viable, with almost no signs of cell death over 7 days of culture. For visualization purposes, the LC were removed from the coacervate and observed separately, then compared to LC cultured independently, without coacervate exposure (LC control) (see Figure 5d). It was evident that, over the 7‐day culture period, the cells within the LC incorporated into the coacervate remained viable, similar to what was observed to the control group. These results showcase the importance of the capsule's semipermeable membrane in ensuring the diffusion of essential molecules, such as oxygen and nutrients, necessary for cell survival [83, 84, 85]. Similarly, the membrane acts as a selective barrier, preventing the exchange of higher molecular weight compounds and shielding the core content from direct exposure to the surrounding coacervate material. Furthermore, DAPI/Phalloidin staining (see Figure 5e) demonstrated that incorporation of the LC into the coacervate did not affect the cells´ ability to adhere to the PCL microparticles, providing additional evidence of cell viability and proliferation.

**FIGURE 6:**
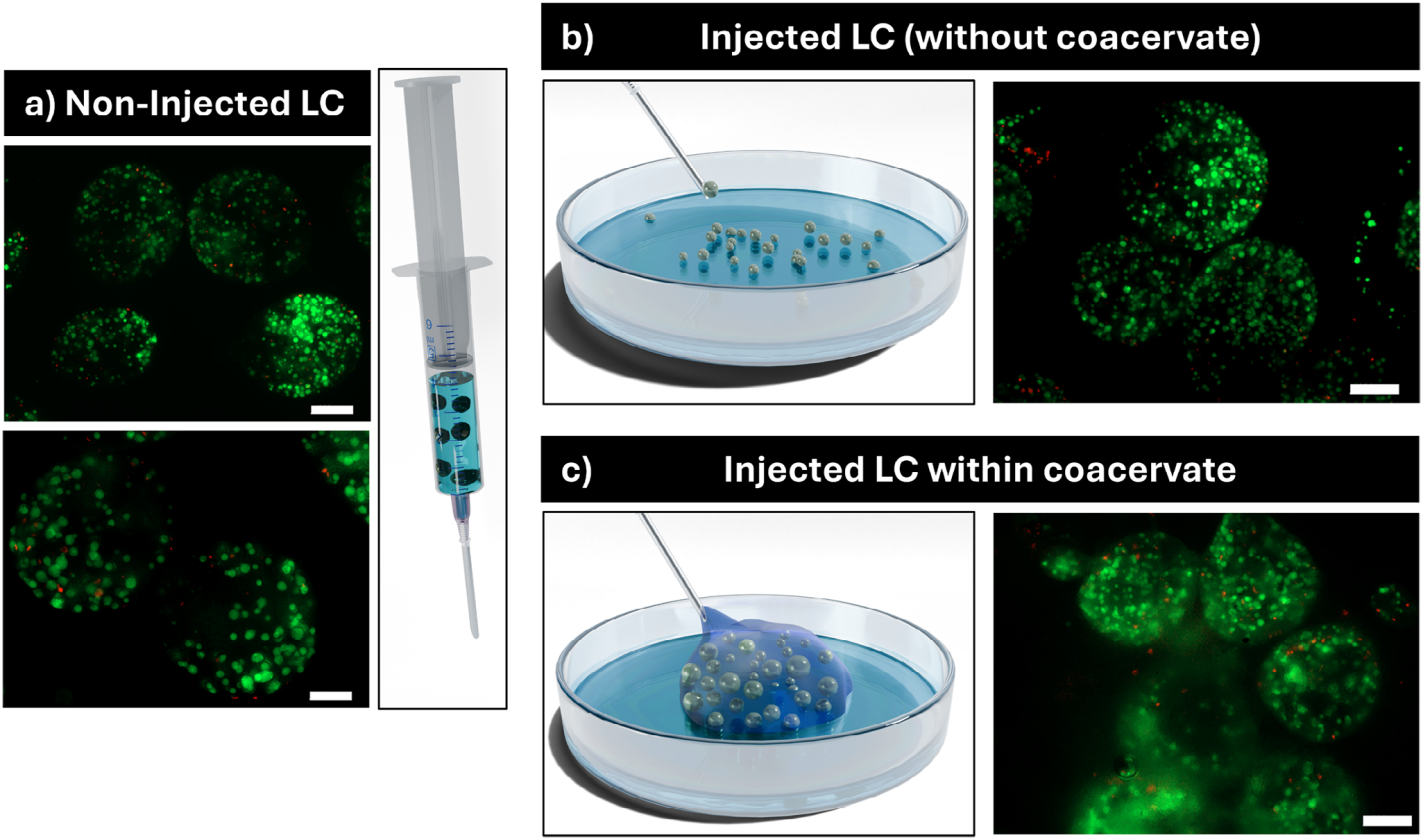
Evaluation of hASCs‐laden LC viability post‐injection. Representative Live/Dead fluorescence images of (a) non‐injected LC (scale bar: top 200 µm; Bottom 100 µm), (b) LC injected alone (scale bar = 200 µm), and (c) LC embedded within the complex coacervate (scale bar = 200 µm). Injections were performed using a standard 21G needle. LC were mixed with the complex coacervate at a volumetric ratio of 54%.

Given that the proposed system is intended for minimally invasive delivery, it is important to evaluate whether the injection process affects the structural integrity of the LC or compromises cell viability. To this end, an injection test was performed using a standard 21G needle (Figure 6). The results show that hASCs‐laden LC embedded within the complex coacervate remain viable, comparable to those in LC injected alone. These findings indicate that the coacervate matrix does not impose significant additional mechanical stress on the capsules during injection and supports their safe delivery. To the best of our knowledge, the ability to encapsulate living entities within complex coacervates assembled at acidic pH is here reported for the first time. We envision the developed platform as a synergistic system, where the transport matrix and the biological units work together to guide tissue regeneration. The complex coacervate provides initial protection and on‐site fixation (see Movie S1), while the cell‐laden LC support paracrine signaling and extracellular matrix deposition [81, 86]. Over time, owing to its dynamic nature, the coacervate gradually remodels facilitating micro‐tissue fusion, enabling eventual integration into regenerating tissue. This technology offers a new conceptual framework for cell integration, paving the way toward more versatile and biologically adaptive materials. However, further in vivo studies are required to fully assess the biocompatibility, immune response, biodegradability, and tissue repair capacity of the proposed system. Nevertheless, the primary components of this biomaterial have been widely reported to exhibit excellent biocompatibility and biodegradability, while also mitigating inflammatory responses, particularly in wound healing applications [33, 87, 88, 89, 90]. Accordingly, based on the present in vitro findings and comparisons with similar systems, it is reasonable to anticipate that this injectable, cell‐laden material holds strong potential for tissue regeneration applications.

## 3 Conclusions

In this work, we report for first time the design of a thermoresponsive complex coacervate assembled via electrostatic interactions between natural polysaccharides grafted with PNIPAAm chains. Upon optimization of the assembly conditions, the resulting coacervates exhibited pronounced shear‐thinning behavior, a key feature for in situ application. Furthermore, the thermally induced structuring of the coacervate was demonstrated both visually and through rheological measurements, showcasing a rapid and complete sol–gel transition at physiological temperature. The “smart” complex coacervate could support the introduction of LC containing cells and microparticles, acting as microtissues incubators. Incorporating LC preloaded with hASCs enabled the coacervates to successfully sustain high cell viability. By combining the ‘smart’, stimuli‐responsive behavior of the coacervates with hierarchically organized, cell‐laden microconstructs, we introduce a novel strategy for the development of injectable materials capable of sustaining cell viability over extended periods. This versatile platform not only enables precise delivery and mechanical integration of bioactive components at defect sites but also opens new avenues for in situ cell delivery systems in tissue engineering, as the LC can be adapted to carry virtually any, or even multiple, cell types. Together, these features highlight the potential of complex coacervate‐based systems to advance regenerative therapies by mimicking the modularity and compartmentalization of native tissues.

## 4 Experimental Section

### 4.1 Reagents and Materials

N‐Isopropylacrylamide (stabilized with MEHQ), 6‐aminofluorescein, and 2,2′‐Azobis(2‐methylpropionitrile) (AIBN) were purchased from Sigma–Aldrich. Prior to use, AIBN was purified by recrystallization in methanol. 2‐Aminoethanethiol (AESH), 3‐mercaptopropionic acid (MPA), 1‐(3‐dimethylaminopropyl)‐3‐ethylcarbodiimide hydrochloride (EDC), and N‐hydroxysuccinimide (NHS) were obtained from TCI Chemicals. Hyaluronic acid sodium salt (80–100 kDa) was purchased from Biosynth. CHT (96 kDa) with a degree of deacetylation (DA) of 97,2% was kindly provided by Primex ehfs (Siglufjordur, Iceland) and used without further purification. Unless otherwise specified, all used reagents were of analytical grade and used as received, without further purification.

### 4.2 Synthesis of ‐NH_2_ and ‐COOH Terminated PNIPAAm

PNIPAAm–NH_2_ and PNIPAAm‐COOH were synthesized by chain transfer polymerization of NIPAM using AIBN as initiator [91, 92]. Briefly, NIPAM (44.19 mmol) was dissolved in DMF (20 mL) following the addition of AESH (2.71 mmol) to obtain PNIPAAm‐NH_2_ or MPA (2.71 mmol) for PNIPAAm‐COOH. The mixture was purged with N_2_ for 30 min prior to the addition of AIBN (7.61 mmol). The polymerization reaction took place at 70°C for 6 h. The reaction mixture was precipitated in cold diethyl ether and the precipitate was filtered and dried under vacuum. The dried polymer was dissolved in cold water and transferred into a dialysis bag (3.5 kDa), dialyzed for 5 days, while the water was changing twice a day. The purified polymer was obtained by freeze‐drying, giving rise to a white foam.

### 4.3 DMF‐Based Size‐Exclusion Chromatography (DMF‐SEC)

DMF‐SEC was conducted on a GPCMax system (Viscotek) equipped with 302 TDA detectors array and two columns in series (PolarGel L and M, both 8 µm 30 cm, Agilent Technologies). The columns and detectors were kept at a temperature of 50°C. DMF containing 0.01 M lithium bromide (LiBr) was used as eluent at a flow rate of 1 mL min^−1^. Near monodisperse poly(methyl methacrylate) (PMMA) standards (Polymer Standard Services) were used as calibrants. Samples were dissolved in the eluent at a concentration of ≈3 mg mL^−1^ and passed through a 0.45 or 0.22 µm PTFE filter prior to injection. Data acquisition and calculations were performed using Viscotek Omnisec software version 5.0.

### 4.4 CHT‐*g*‐PNIPAAm and HA‐*g*‐PNIPAAm Grafting

EDC/NHS coupling chemistry was employed to graft the PNIPAAm chains onto CHT and HA backbone. CHT (1.0 g) was dissolved in 90 mL of MiliQ ultrapure water, and the pH was adjusted to 5.5, using 0.1 m NaOH or HCl. Separately, EDC (19.2 mg, 0.1 mmol) and NHS (11.5 mg, 0.1 mmol) were added in a 15 min interval to a PNIPAAm‐COOH (0.4 g, 0.01 mmol) aqueous solution (10 mL) and stirred until complete dissolution. Afterward, this mixture was added to the CHT solution, and the reaction was stirred overnight at RT. The reaction mixture was transferred into a dialysis bag (10 kDa) and dialyzed for 2 days, while the water was changing twice a day. The mixture was freeze‐dried, and the white foam was washed three times with acetone and centrifuged (10 min, 2000 *g*) to remove any traces of ungrafted PNIPAAm. Finally, the precipitate was dried under vacuum and dissolved in water, transferred into a dialysis bag (10 kDa), and dialyzed for 3 days, while the water was changing twice a day. The purified polymer was obtained by freeze‐drying, giving rise to a white foam with a grafting yield of 49%.

For the synthesis of HA‐*g*‐PNIPAAm, HA (1.0 g) was dissolved in 100 mL of MiliQ ultrapure water, and the pH was adjusted to 5.5, using 0.1 m NaOH or HCl. After complete dissolution, EDC (19.2 mg, 0.1 mmol) and NHS (11.5 mg, 0.1 mmol) were added in a 15 min interval. Last PNIPAAm‐NH_2_ (0.5 g, 0.01 mmol) was added and the reaction was stirred overnight at RT. The purification procedure was the same as for the CHT‐*g*‐PNIPAAm, with a grafting yield of 58%.

### 4.5 Proton (^1^H) Nuclear Magnetic Resonance (NMR) Spectroscopy

All NMR spectra were recorded on an Avance II 300 MHz spectrometer (Bruker, Germany) at 300.13 MHz. Detailed information on the determination of the grafting yield and on the molar ratio between the PE and PNIPAAm can be found in the Supporting Information.

### 4.6 Lower Critical Solution Temperature

The determination of the LCST was performed by analyzing the transmittance at 480 nm, as the temperature increased from 25°C to 45°C with a 5°C/min scan speed. Each sample was prepared at 1 mg/mL and transferred to a quartz cuvette. The transmittance was recorded in a GBC Cintra 303 (GBC Scientific Equipment, Australia) spectrophotometer coupled with a Peltier‐controlled thermo‐electric cell holder. The LCST was determined as a 50% transmittance decreased from the initial transmittance.

### 4.7 Optical Mixing Conditions

#### 4.7.1 pH

To achieve a higher yield of coacervation, it is important to mix the two PE at an optimal pH at which both HA‐*g*‐PNIPAAm and CHT‐*g*‐PNIPAAm exhibit the highest degree of ionization. With this in mind, we evaluate the optimal mixing pH by conducting zeta potential measurements. Solutions of each PE (1 mg/mL) were prepared in a pH range from 2.5 to 6.5, and their respective zeta potential was measured. To determine the pH value that maximizes electrostatic interactions between the oppositely charged PE, the strength of electrostatic interactions (SEI) was plotted as a function of pH [13]. SEI values at each pH were calculated as the product of the absolute values of the zeta potential of both PE. Last, a third order polynomial line was fitted to the data to determine the pH at which electrostatic interaction is strongest.

#### 4.7.2 Polyelectrolyte Ratio

To determine the optimal mass mixing ratio, stock solutions (10 mg/mL) of CHT‐g‐PNIPAAm and HA‐g‐PNIPAAm were prepared separately in 1% (v/v) acetic acid and MiliQ ultrapure water, respectively. While under magnetic stirring, the two solutions were mixed in ratios ranging from 0.25 to 2 (CHT‐*g*‐PNIPAAm:HA‐*g*‐PNIPAAm). After 20 min, the mixture was collected and centrifugated at 2000 *g* for 10 min. Upon centrifugation, the diluted phase was collected, and its zeta potential was measured. The optimal mixing ratio was defined as the mass ratio at which the diluted phase has a zeta potential charge near 0, indicating effective charge balance and minimal excess charge in the diluted phase.

#### 4.7.3 Salt Concentration

Turbidity served as a qualitative measure to assess the degree of coacervation, which is influenced by both charge stoichiometry and salt concentration [46]. Accordingly, turbidity measurements (expressed as O.D.) were performed at 550 nm using a Synergy HTX multi‐mode microplate reader (BioTek Instruments, Winooski, VT). Coacervates were prepared as previously described (pH 5, 1:1 ratio), while varying the NaCl concentrations, ranging from 10 to 500 mm. Last, 200 µL of the supernatant from each condition was transferred to a 96‐well plate for analysis (*n = 3*).

### 4.8 Complex Coacervation Formation

Stock solutions (10 mg/mL) of CHT‐*g*‐PNIPAAm and HA‐*g‐*PNIPAAm were prepared separately in 1% (v/v) acetic acid and Mili‐Q ultrapure water, respectively. The pH of both solutions was adjusted to 5, using 0.1 m NaOH or HCl. Last, NaCl was added to a final concentration of 100 mm from a 1 m stock solution. While under constant magnetic stirring, the two solutions were mixed in a 1:1 volumetric ratio, leading to immediate complexation. After 20 min of stirring at 300 rpm, the mixture was collected and centrifugated at 2000 *g* for 10 min. Upon centrifugation, the polymer‐rich phase (coacervate), deposited at the bottom of the centrifuge tube, was collected (final pH ≈ 5), while the diluted phase (supernatant) was discarded unless further analysis needed to be performed. Native complex coacervates, formed from the native PE (without grafted PNIPAAm), were obtained by following the same protocol.

### 4.9 Water Content

The water content of the coacervate below and above the LCST (25°C and 37°C) was determined as previously reported [72]. After the formation of the complex coacervates, 80 µL samples were weighed (W_wet_), then the samples were freeze‐dried and their dry weight (W_dry_) was recorded. The water content was calculated as follows:

(1) $$Water\;content\left(\%\right)=\left(\frac{W_{\mathrm{wet}}-W_{\mathrm{dry}}}{W_{\mathrm{wet}}}\right)\times 100$$

To determine the water content at 37°C, coacervates were first incubated in phosphate‐buffered saline (PBS, Sigma–Aldrich) at 37°C for 15 min, carefully dried with paper to remove any superficial water, and their W_wet_ was recorded.

### 4.10 Swelling (%)

The swelling percentage of the coacervate was evaluated after 1, 5, 15, 30, and 60 min of incubation in PBS at 37°C by measuring the change in wet weight (*W_t_*) at each timepoint relative to the initial weight (*W*_0_), according to the following equation:

(2) $$Swelling\left(\%\right)=\frac{W_{t}-W_{0}}{W_{0}}\times 100$$

### 4.11 Rheological Characterization

The coacervates were characterized using a Kinexus Lab+ rotational rheometer (Malvern PANalytical, U.K.) equipped with an 8 mm diameter parallel plate geometry, with the gap fixed at 0.5 mm. Oscillatory strain amplitude sweep measurements at a fixed frequency (1 Hz) were conducted at 25°C to determine the linear viscoelastic region (LVER). Further tests were performed at 0.1% strain (within the LVER). The viscoelastic behavior was further evaluated by assessing the G′ and G″ through oscillatory frequency sweep tests at both 25°C and 37°C. The stress relaxation experiment was performed at constant 10% strain. A temperature sweep assay from 25°C to 37°C, at a rate of 2°C/min was performed to study the G' profile with increasing/decreasing temperature. Additionally, the evolution of G' was analyzed at a constant temperature of 37°C. The shear‐thinning profile of the coacervates was analyzed by studying the shear‐viscosity (Pa.s) across increasing shear‐rate (s^−1^). A power‐law model was fitted to the experimental data using the following equation:

(3) $$\eta =k{\dot{\gamma }}^{n-1}$$

where η is the shear viscosity (Pa.s^−1^), k is the consistency index, γ is the shear rate, and *n* is the flow index.

### 4.12 Production and Surface‐Functionalization of PCL Microparticles

Polycaprolactone (PCL) microparticles were produced by emulsion solvent evaporation technique as described elsewhere [84]. Briefly, a 5% (w/v) PCL (molecular weight (Mw) ∼ 80 000, Merck) solution prepared in methylene chloride (Honeywell) was slowly added to a stirring 0.5% (w/v) polyvinyl alcohol (PVA, Merck) solution prepared in distilled water. After 2 days under stirring at room temperature (RT), PCL microparticles were collected, washed several times with distilled water, and sieved to obtain a diameter range of 40–50 µm. Afterward, the microcarrier's surface was functionalized by plasma treatment technique in a low‐pressure plasma reactor chamber (ATTO, Diener Electronic) at 30 V and 0.2–0.4 mbar for 15 min. The microparticles were sterilized in 70% v/v ethanol for 2 h. Finally, PCL microparticles were coated with collagen I (10 µg/cm^2^, rat protein tail, ThermoFisher Scientific) at RT overnight.

For visualization purposes, a coumarin 6 solution (2 mg/mL in acetone, Sigma–Aldrich) was prepared, and 70 µL of this solution was added to 8 mL of a 5% (w/v) PCL solution. The production of coumarin 6–stained PCL microparticles was carried out as described above.

### 4.13 Cell Culture

To assess the in vitro biological performance, two cell types were used: L929 mouse fibroblasts (ATCC CRL‐6364), as recommended by the ISO 10993 standard for direct contact biocompatibility testing, and human adipose tissue‐derived mesenchymal stem cells (hASCs) (ATCC PCS‐500‐011), as an adherent cell type for encapsulation within the LC. L929 fibroblasts were cultured in T‐175 tissue culture flasks (Sarstedt) containing low glucose Dulbecco's Modified Eagle Medium (DMEM) supplemented with 10% (v/v) fetal bovine serum (FBS, ThermoFisher Scientific) and 1% (v/v) antibiotic/antimycotic solution (penicillin/streptomycin, ThermoFisher Scientific), under standard conditions (37°C, 5% CO_2_). Once confluent, the cells were detached using trypsin‐EDTA (ThermoFisher Scientific) and seeded at a density of 1.0 × 10^4^ cells/well for 24 h prior to the assay. hASCs were cultured in T‐175 tissue culture flasks with Minimum Essential Medium alpha (α‐MEM, Sigma–Aldrich), supplemented with 10% (v/v) FBS and 1% (v/v) antibiotic/antimycotic, under standard conditions. Once confluent, the cells were detached using trypsin‐EDTA and encapsulated in LC.

### 4.14 Cell Viability Assays

For the L929 assay, freshly prepared coacervates (80 µL) were placed in direct contact with the seeded cells and incubated for 1, 5, and 7 days. As a control, cells seeded at the same density were cultured without coacervate exposure. At each time point, cell viability was assessed using a Live/Dead assay. Cells were incubated at 37 °C for 20 min in Dulbecco's Phosphate Buffered Saline (DPBS, Sigma–Aldrich) solution containing calcein‐AM (2 µg/mL, ThermoFisher Scientific) and propidium iodide (PI, 1 µg/mL, ThermoFisher Scientific), protected from light. Stained samples were imaged using a Zeiss Imager M2 widefield fluorescence microscope equipped with a 3‐megapixel camera (Carl Zeiss Microscopy, Oberkochen, Germany). Image analysis was performed using ZEN v2.3 Blue Edition software (Zeiss).

In parallel, the metabolic activity of L929 cells was evaluated using the AlamarBlue Cell Viability Reagent (Alfagene, Lisbon, Portugal). Following medium removal, a 10% (v/v) AlamarBlue solution in DMEM was added and incubated for 4 h at 37°C. Fluorescence was measured with a Synergy HTX multi‐mode microplate reader (BioTek Instruments, Winooski, VT) at excitation/emission wavelengths of 570/585 nm. For the hASC‐laden LC, cell viability was assessed using the same Live/Dead staining protocol. At each time point, LC incorporated in the coacervate (54% volumetric fraction), and LC‐only controls were incubated with calcein‐AM and PI for 20 min at 37°C, protected from light, and imaged under identical conditions.

### 4.15 DAPI/Phalloidin Staining

To analyze cell morphology, cells were stained with phalloidin (1:40 (v/v) in DPBS, Flash Phalloidin Red 594, BioLegend) at RT for 20 min, followed by incubation with DAPI (1:1000 (v/v) in DPBS, Thermo Fisher Scientific), for 5 min at RT, protected from light. Stained samples were imaged using a Zeiss Imager M2 widefield fluorescence microscope equipped with a 3‐megapixel camera (Carl Zeiss Microscopy, Oberkochen, Germany). Image analysis was performed using ZEN v2.3 Blue Edition software (Zeiss). Proliferation assay analysis was performed using DAPI‐stained images, with cell density (cells/mm^2^) determined by nuclear counting through ImageJ analysis. For the hASC‐laden LC, cell morphology was assessed using the same DAPI/phalloidin staining protocol described above.

### 4.16 Cell‐Laden Liquid‐Core Capsules Production

LC were produced via interfacial complexation of two oppositely charged polyelectrolytes in an all‐aqueous two‐phase system (ATPS) through electrohydrodynamic atomization (EHDA) technique (Spraybase, Avectas), as described elsewhere [81]. Briefly, a Phase I solution composed of 15% (w/w) Dextran (DEX) from *Leuconostoc spp*. (Mw ≈ 500 kDa, Sigma–Aldrich) and 1% (w/w) alginic acid sodium salt (ALG, Sigma–Aldrich) (PBS, Sigma–Aldrich). Phase II solution comprised 17% (w/w) polyethylene glycol (PEG, Mw ≈ 8 kDa, Sigma–Aldrich) and 0.5% (w/w) ε‐poly‐L‐lysine (PLL, Mw ≈ 4.7 kDa, Epolyly Pure, Handary S.A.), also prepared in PBS and sterilized. A suspension of hASCs (3.0 × 10^6^ cells/mL) and surface‐functionalized PCL microparticles (30 mg/mL) were resuspended in Phase I solution (DEX/ALG). Cell‐laden liquid‐core microcapsules were then generated by EDHA technique, using phase II (PEG/PLL) as collecting bath. After 5 min of complexation, microcapsules were washed three times in cell medium. Finally, LC were cultured in a spinner flask (Celstir, Wheaton) with α‐MEM, supplemented with 10% (v/v) FBS and 1% (v/v) antibiotic/antimycotic for 24 h to allow cell adhesion to PCL microparticles.

### 4.17 Complex Coacervates Loaded With LC

Freshly prepared coacervates were assembled as described above. After centrifugation, several LC densities were selected and mixed with the coacervate. Subsequently, repeated up‐and‐down cycles using a micropipette were performed to homogenize the mixture. The volumetric ratios were calculated by dividing the total volume of LC capsules, based on their average diameter (469 ± 69 µm), by the total volume of coacervate used. For visualization purposes, the PCL microparticles within the LC were stained with coumarin 6, as described above.

### 4.18 Statistical Analysis

All data were statistically evaluated resorting to GraphPad Prism software (version 10.1.0) and reported as mean ± standard deviation (*n* = 3). Statistical differences were considered significant when the *p*‐ value < 0.05, established by an unpaired *t*‐test.

## Author Contributions

L.P.G.M. – formal analysis, experimental designs, investigation, methodology, writing – original draft; M.C. – investigation (liquid‐core capsules, in vitro studies), writing – review & editing; J.E.S. – investigation (complex coacervation characterization), writing – review & editing. M.K. – conceptualization, supervision, resources, writing – review & editing; J.M.M.R. – conceptualization, methodology, supervision, funding, writing – review & editing. J.F.M. –conceptualization, supervision, resources, funding, writing – review & editing. All authors analyzed the data, discussed the results, and have given approval to the final version of the manuscript.

## Conflicts of Interest

The authors declare no conflicts of interest.

## Supporting information




**Supporting File**: smll73410‐sup‐0001‐SuppMat.pdf.

**Supporting File**: smll73410‐sup‐0002‐MovieS1.mp4.

## Data Availability

The data that support the findings of this study are available from the corresponding author upon reasonable request.
