# Supplementary material for: Thermoresponsive Complex Coacervates as Advanced Carriers for Cell‐Laden Liquid‐Core Capsules for Biomedical Applications
Source: Small. 2026 Apr 16;22(32):e13642. doi: 10.1002/smll.202513642 (PMC13244403; doi:10.1002/smll.202513642)
Supplement: Supplementary file 1 — Supporting File: smll73410‐sup‐0001‐SuppMat.pdf. [file SMLL-22-e13642-s001.pdf]

Supporting information

Thermoresponsive complex coacervates as advanced carriers for cell-laden liquid-core capsules for biomedical applications

Luís P. G. Monteiro [1], Mariana Carreira [1], Julien Es Sayed [2], Marleen Kamperman [2], João M. M. Rodrigues *[1], João Mano *[1]

[1] CICECO – Aveiro Institute of Materials, Department of Chemistry, University of Aveiro, 3810-193 Aveiro, Portugal

[2] Zernike Institute for Advanced Materials, University of Groningen, Nijenborgh 3, 9747 AG Groningen, the Netherlands.

* Corresponding authors: jrodrigues@ua.pt; jmano@ua.pt

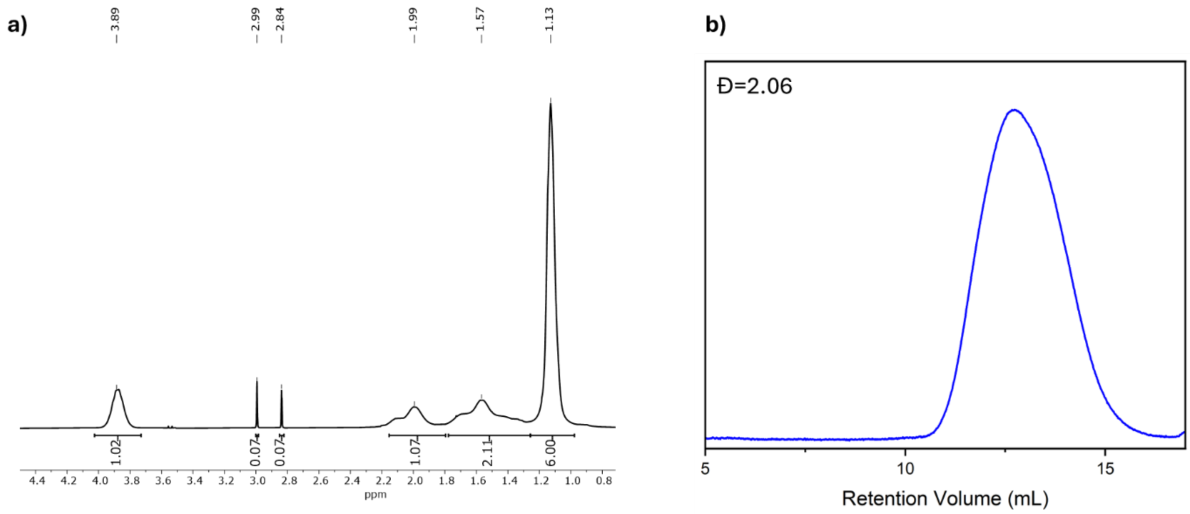

Figure S1: a) ^{1}H NMR spectrum of PNIPAAm-NH_2 in D_2O and (b) corresponding SEC elution profile recorded in DMF containing 0.01 M LiBr.

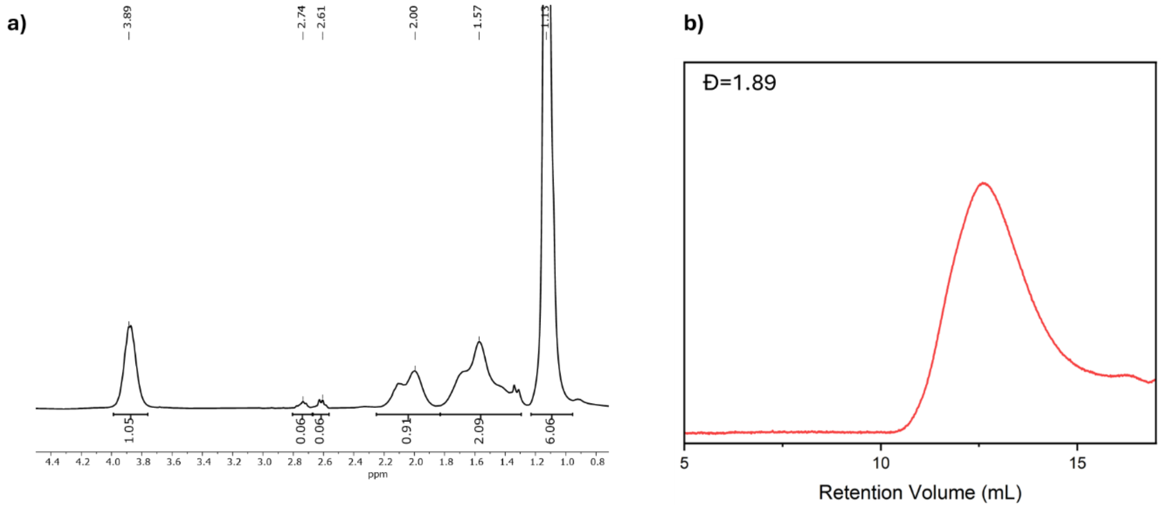

Figure S2: a) ^{1}H NMR spectrum of PNIPAAm-COOH in D_2O and (b) corresponding SEC elution profile recorded in DMF containing 0.01 M LiBr.

Molar ratio and grafting yield

The molar ratio of PNIPAAm chains was calculated based on the ^{1}H NMR spectra as follows [1]. The integral of the two CH_3 groups of PNIPAAm (I_{PNI}) was set to 6 (6H). Next, due to the possible signal overall between the PE methyl group and protons of the PNIPAAm chain backbone, the peak area between 1.2 and 2.2 ppm (I_{PE}) was subtracted by 3.0 (number of

protons belonging to the PNIPAM backbone) and divided by 3.0 (number of protons belonging to the PE methyl group). Lastly the molar ratio was obtained as follows:

$$NIPAAm\ molar\ ratio = \left(1 - \frac{I_{PE}}{I_{PE} + \frac{I_{PNI}}{6}}\right) \times 100$$

The grafting yield was determined as follows [2]:

$$Grafting\ \text{yield (\%)} = \left(\frac{W_2 - W_1}{W_1}\right) \times 100$$

where W_2 is total weight obtained after the reaction, and W_1 is the initial weight of HA/CHT used in the synthesis.

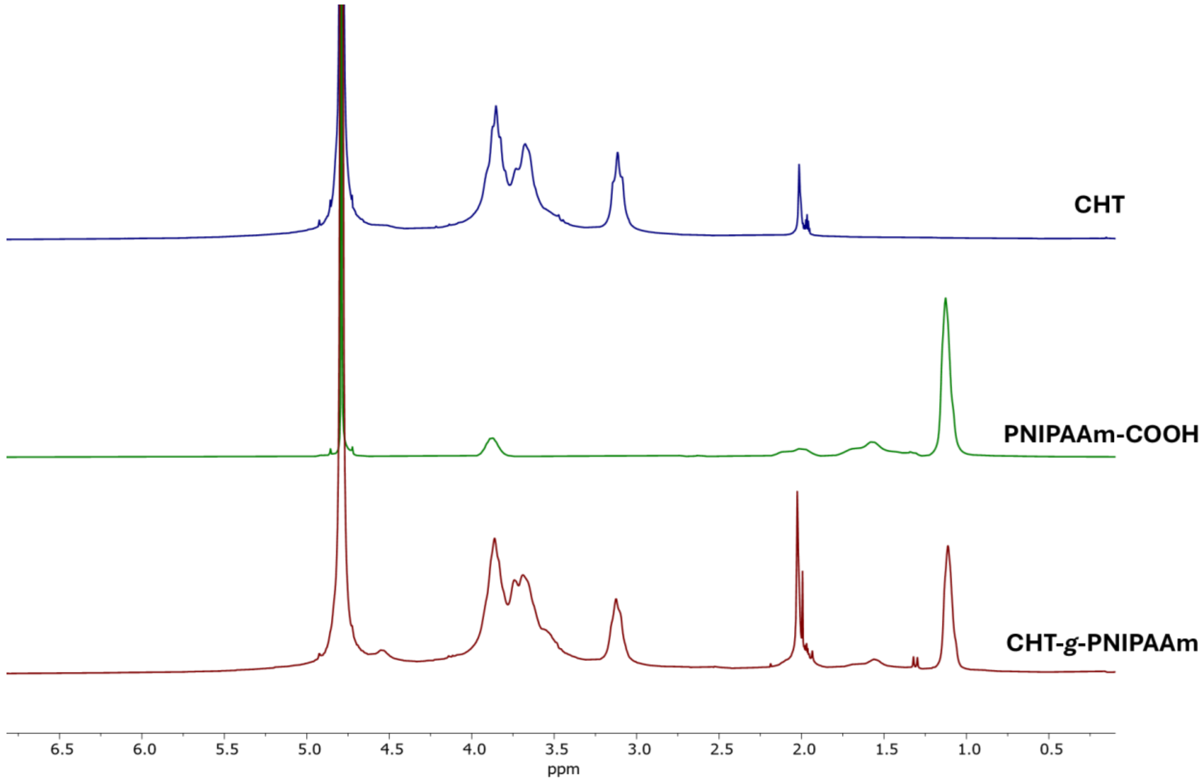

Figure S3: ^{1}H NMR spectra recorded in D_2O of CHT-*g*-PNIPAAm (red), native CHT (blue), and PNIPAAm-COOH (green)

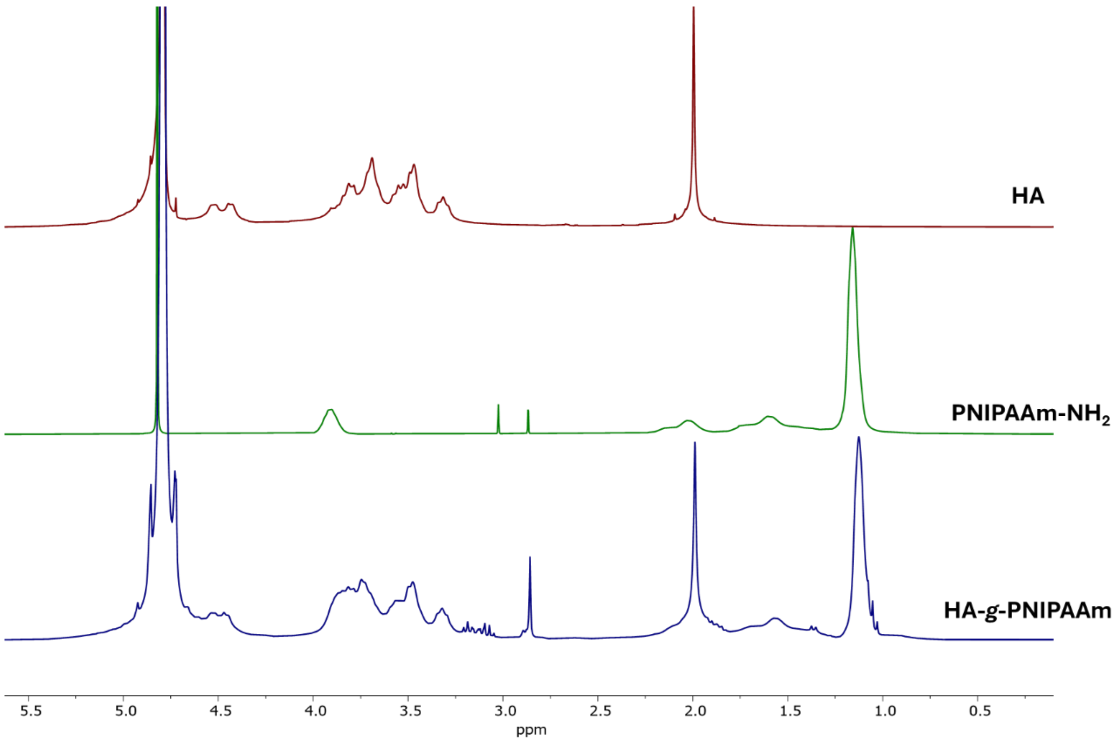

Figure S4: ^{1}H NMR spectra of HA-*g*-PNIPAAm (blue), native HA (red), and PNIPAAm-NH_2 (green) recorded in D_2O.

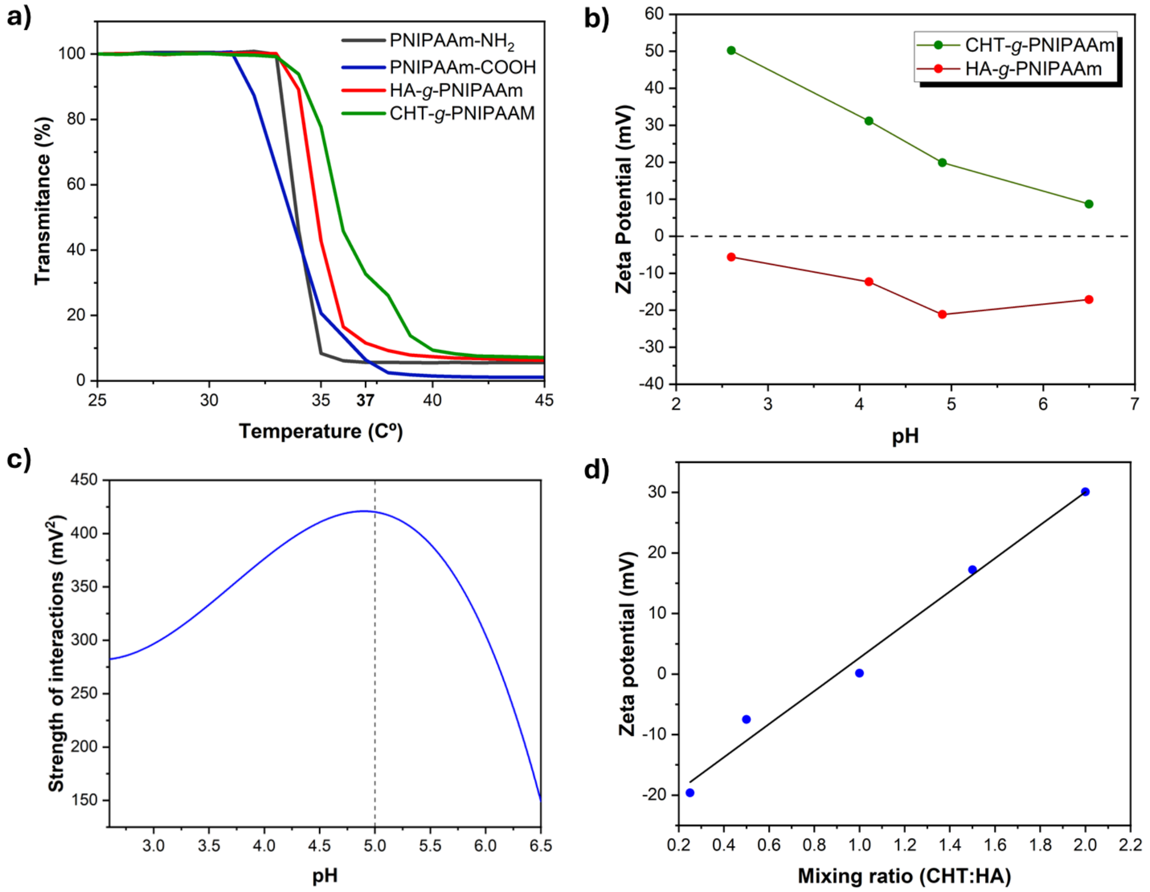

Figure S5: a) Turbidity analysis (% transmittance) to determine the LCST by UV–Vis absorbance with increasing temperatures; b) Zeta potential (mV) of PNIPAAm-grafted PE as a function of pH and c) corresponding strength of electrostatic interactions (SEI). d) Zeta potential (mV) of the diluted phase of the coacervates as a function of the mass mixing ratio.

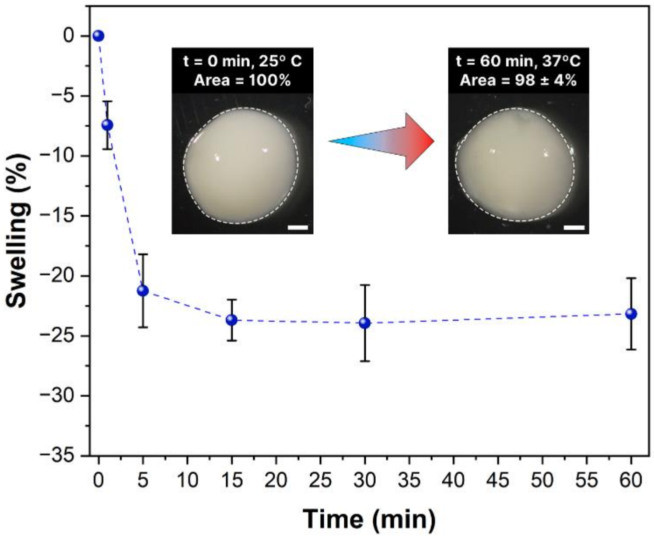

Figure S6: Swelling (%) of the coacervate over time following exposure to 37°C. Quantification of the macroscopic shrinkage, calculated from the reduction in the projected area of the coacervate using ImageJ (inset). Scale bar = 1000 µm.

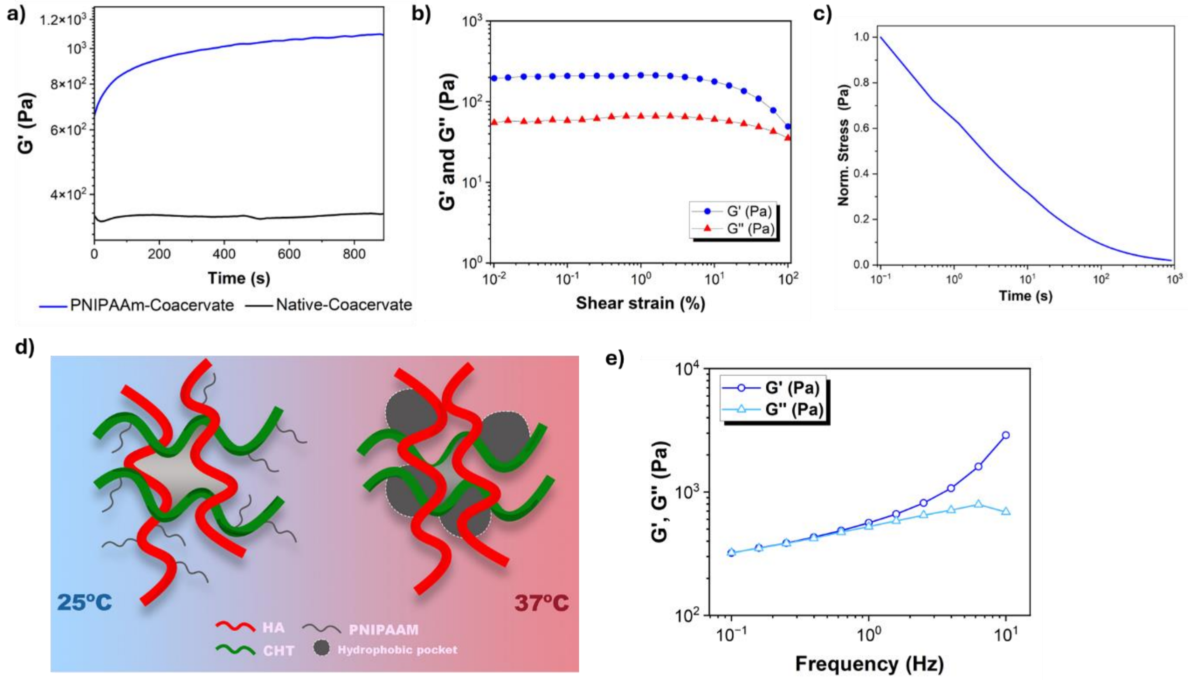

Figure S7: a) Time-sweep test at 37°C comparing the native and thermoresponsive coacervates under fixed frequency (1 Hz) and strain (0.1%); b) Strain-sweep test (0.01–100%) at constant frequency (1 Hz) performed to determine the linear viscoelastic region (LVER) of the thermoresponsive coacervate, identified between 0.01 and 2.5% strain. c) Stress relaxation profile at constant strain (10%) for the thermoresponsive coacervate preformed at

25°C. d) Schematic representation illustrating the enhanced packing of PE chains at 37°C, driven by PNIPAAm aggregation above its LCST. e) Frequency sweep of the thermoresponsive coacervate performed after incubation at 37°C for 30 min followed by equilibration at 25°C for 1 h. Measurements were performed within the linear viscoelastic region.

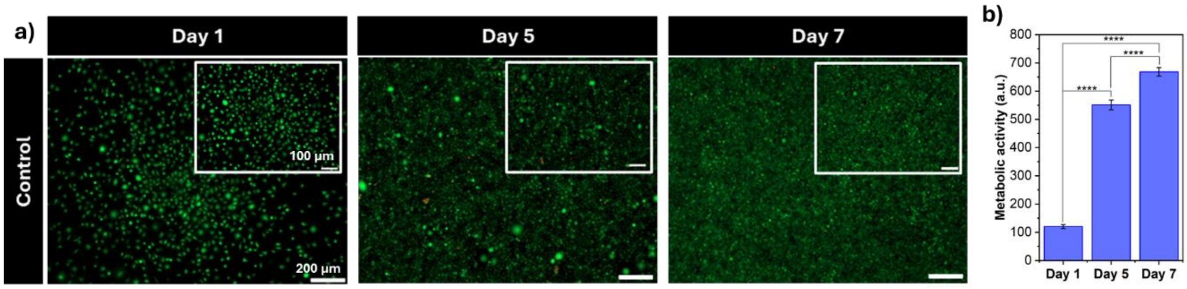

Figure S8: a) Live/dead fluorescence microscopy images of L929 cells culture in the absence of coacervate (control). Staining includes Calcein AM (green, live cells) and propidium iodide (PI, red, dead cells). b) Quantitative analysis of cellular metabolic activity at the corresponding time points (n = 3). p-value ≤ 0.0001 (****).

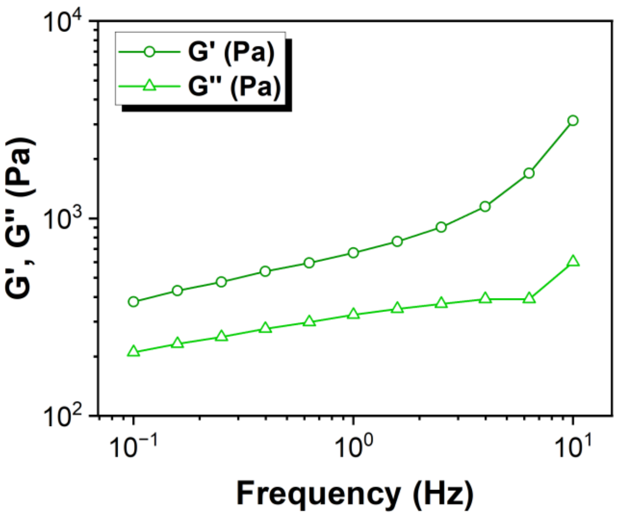

Figure S9: Frequency sweep of the thermoresponsive coacervate after incubation in PBS (pH 7.4) for 30 min, showing the G′ and G″ (Pa) as a function of frequency (Hz). Measurements were performed within the linear viscoelastic region.

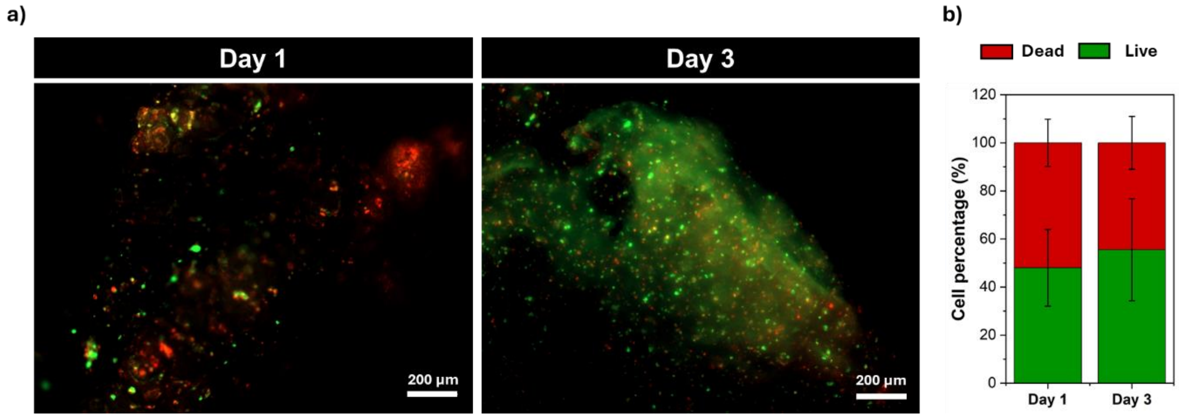

Figure S10: a) Live/dead fluorescence microscopy images of hASCs cells directly encapsulated into the coacervate (1.0×10^6 cells/mL) and cultured for 1 and 3 days. Staining includes Calcein AM (green, live cells) and propidium iodide (PI, red, dead cells). b) Quantitative fluorescence quantification of live (green) and dead (red) cells performed using ImageJ (*n* = 3).

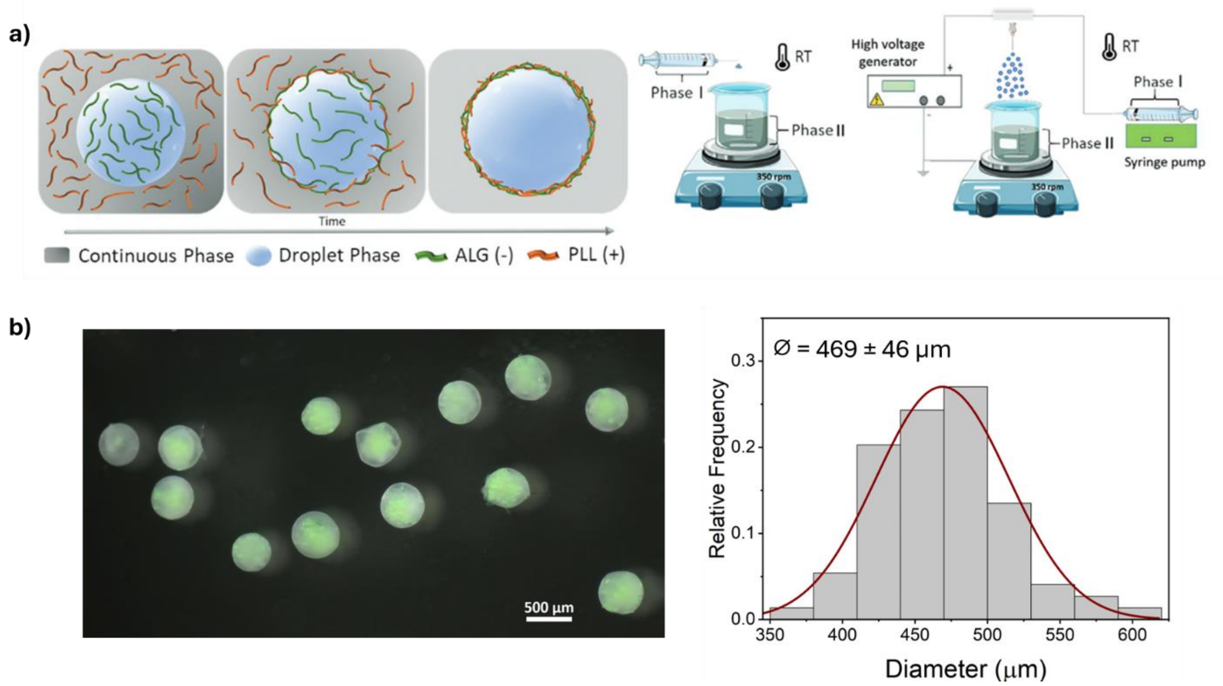

Figure S11: a) Schematic illustration of the setup used for the electrostatic assembly of the LC in an all-aqueous environment, employing electrohydrodynamic atomization equipment, as previously reported by our group [3]. Copyright © 2021 Wiley-VCH GmbH. b) Digital image of the LC loaded with fluorescent PLC microcarriers and the corresponding diameter distribution histogram.

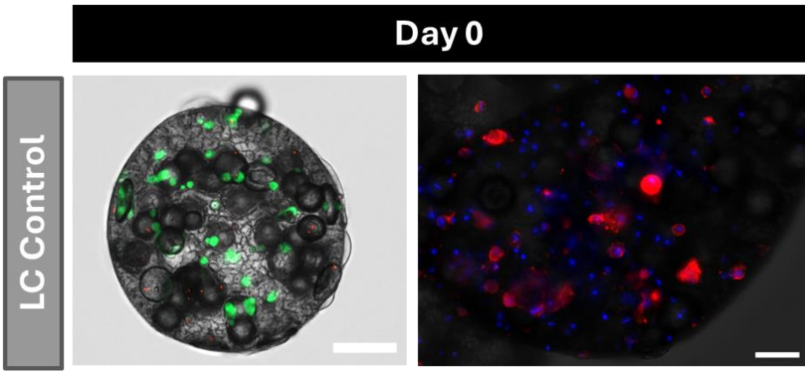

Figure S12: Live/Dead (scale bar = 100 µm) and DAPI/phalloidin (scale bar = 50 µm) fluorescence staining of hASCs encapsulated within the LC prior to coacervate loading.

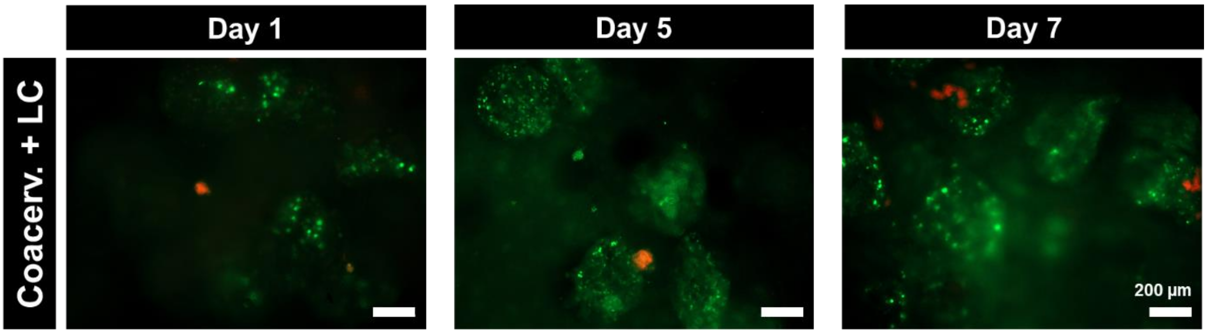

Figure S13: a) Live/dead fluorescence microscopy images of thermoresponsive coacervates loaded with hASCs-laden LC (54% volumetric fraction).

References:

1. M. Dompé, F. J. Cedano-Serrano, O. Heckert, et al., "The Essential Role of the Electrolyte–Electrode Interface for the Stability of All-Solid-State Lithium Batteries," *Advanced Materials* 31, no. 21 (2019): 1808179, https://doi.org/10.1002/adma.201808179
2. D. I. Ha, S. B. Lee, M. S. Chong, Y. M. Lee, S. Y. Kim, and Y. H. Park, "Preparation of Thermo-Responsive and Injectable Hydrogels Based on Hyaluronic Acid and Poly(N-isopropylacrylamide) and Their Drug Release Behaviors," *Macromolecular Research* 14, no. 1 (2006): 87–93, https://doi.org/10.1007/Bf03219073
3. S. Vilabril, S. Nadine, C. Neves, et al., "One-Step all-Aqueous Interfacial Assembly of Robust Membranes for Long-Term Encapsulation and Culture of Adherent Stem/Stromal Cells," *Advanced Healthcare Materials* 10, no. 10 (2021): 2100266, https://doi.org/10.1002/adhm.202100266
